# Application of Powder-Bed Fusion of Metals Using a Laser for Manufacturing of M300 Maraging Steel Tools Intended for Sheet Metal Bending

**DOI:** 10.3390/ma17246185

**Published:** 2024-12-18

**Authors:** Krzysztof Żaba, Maciej Balcerzak, Łukasz Kuczek, Marcel Wiewióra, Ilona Różycka, Tomasz Trzepieciński, Jarosław Mizera

**Affiliations:** 1 Department of Metal Working and Physical Metallurgy of Non-Ferrous Metals, Faculty of Non-Ferrous Metals, AGH University of Krakow, Al. Adama Mickiewicza 30, 30-059 Cracow, Poland; krzyzaba@agh.edu.pl (K.Ż.); balcerzak@agh.edu.pl (M.B.); lukasz.kuczek@agh.edu.pl (Ł.K.); 2 ERKO sp. z o.o. sp.k., Czeluśnica 80, 38-204 Jasło, Poland; marcel.wiewiora@erko.pl; 3 Department of Materials Science and Engineering of Non-Ferrous Metals, Faculty of Non-Ferrous Metals, AGH University of Krakow, Al. Adama Mickiewicza 30, 30-059 Cracow, Poland; rozycka@agh.edu.pl; 4 Department of Manufacturing Processes and Production Engineering, Rzeszów University of Technology, 8 Powstancow Warszawy Ave., 35-959 Rzeszow, Poland; tomtrz@prz.edu.pl; 5 Faculty of Materials Science and Engineering, Warsaw University of Technology, Woloska 141, 02-507 Warsaw, Poland

**Keywords:** laser powder bed fusion, Inconel alloy, aluminium alloy, M300 maraging steel, sheet metal forming, bending tool

## Abstract

This paper presents the results of a pilot application of Powder-Bed Fusion of Metals Using a Laser (PBF-LB/M) for the fabrication of M300 (1.2709) maraging steel sheet metal bending tools. S235 steel was used as a substrate for the fabrication of bending punches. The main goal of the research was to determine the usability of such tools without heat treatment, which would contribute to the increase in the cost of tool production. Industrial tests of tools were conducted during the forming of Inconel 625 and AW-6061 T0 aluminium alloy sheets. The punches were subjected to tests of surface roughness, hardness, microstructure, porosity, and geometric quality in order to verify the quality and accuracy of tools made by the PBF-LB/M technique before and after experimental investigations in industrial conditions in a selected manufacturing company. It was found that tools with an M300 steel working layer after the PBF-LB/M process without heat treatment show suitability for bending sheet metal in a certain range of force parameters, ensuring obtaining elements after bending from Inconel 625 and AW-6061 T0 aluminium alloy sheets of the required geometric quality.

## 1. Introduction

Currently, we can observe a shift from mass production to personalised mass production. Changing methods of production require alternative manufacturing techniques and processes to meet individual customer needs in terms of the cost and time consumption of large-scale industrial production, as a result of which, alternative approaches are inevitable [1]. Due to the pervasive use of the sheet metal stamping process, which helps in manufacturing complex drawpieces and trends related to the growing demand for faster production lead times in the automotive and aerospace industries, there is an urgent need for a cost-effective method of manufacturing dies and punches for sheet metal forming [2,3]. The tool sector is the foundation of the machine-building industry. Tooling has a significant impact on the quality of final products and components; therefore, the requirements in this area are also increasing [4,5]. Due to the manufacturing related to demanding materials and ensuring the highest durability of its products, the industry is constantly looking for new approaches [6]. Also taking into account the current global economic and geopolitical scenario, the required acceleration of product introduction to the market cannot occur without a significant reduction in tool preparation time [7,8]. It is becoming reasonable to seek answers to the questions of whether and to what extent the use of additive manufacturing methods will accelerate the production of tools in comparison to conventional methods [9]. Commercially available tooling offered by manufacturers is adapted to standard component geometries. However, this option does not always meet the industrial requirements when it comes to creating a tool with a non-standard shape. The need to use an unconventional tool often manifests itself at the initial design stage, as well as after testing the prototype [10]. This requires a dedicated prototyping department that may not be readily available. This disrupts the design and production process, leading to additional costs [11].

Large dies for the production of responsible aircraft components can cost, on average, about 10% of the total cost of the production process [12]. For this reason, the remanufacture of forging and stamping tools and development of the repair procedures are attracting increasing industrial interest. Remanufacturing is a material-saving production model with high benefits for the sustainable development of the industry [13]. One of the leading remanufacturing processes is the Additive Layer Manufacturing process, also known as Laser Metal Deposition (LMD) [14]. The advantage of LMD over conventional welding methods is that metal can be deposited with lower dilution [15]. Foster et al. [16] concluded that LMD with powder can be successfully used for the remanufacturing of high-load forging tools. Pellin et al. [17] developed a procedure to recover steel stamping punches using a gas metal arc welding process and confirmed the performance of this process in repairing the damaged surfaces.

There are many types of manufacturing processes available in the industry, which allow for the production of different types of parts. The classification can be made based on various criteria. The division based on the processing method is extremely important. Plastic forming is a fundamental method of chipless processing [18]. The plastic forming of metals and their alloys occurs by exerting pressure with a tool, which consequently causes exceeding the yield point of the workpiece [19,20,21]. Selecting the proper materials for plastic processing tools is a serious technological problem, primarily due to the variety of their working conditions. These conditions are determined by, among other things, temperature, the value of transferred pressures, the method of loading, the processing speed, and the mass and shape of the tools [22,23]. The problem of ensuring the required tool durability is, therefore, associated not only with the need to ensure the appropriate quality of tool materials and their heat treatment, but also with the selection of the proper material for the specific tool [24,25]. Increasing the efficiency of stamping tools by increasing their durability, efficiency, and reliability remains one of the main challenges for increasing production efficiency [26]. The application of wear-resistant coatings [27] and thermochemical treatment [28] are the most commonly used techniques to improve tool reliability.

Additive manufacturing (AM) processes, also known as rapid prototyping, refer to the evolutionary production of a given component [29,30]. The initiator of additive technology was Chuck W. Hull, who, in 1984, patented the first commercial 3D printing machine SLA-1. The 3D printing method involves reproducing a digital model of the component. The prepared geometric model is cut into different layers, and on this basis, the data for the 3D printer are prepared. The AM of metal components shorten the lead time and cycle time in parts production [31]. AM techniques use solid or liquid materials as the primary medium [32]. There are many benefits that additive manufacturing processes bring. Unfortunately, many of them are not yet available on a laboratory scale and are not suitable for commercial applications in their current form [32,33,34]. Powder-Bed Fusion of Metals Using a Laser (PBF-LB/M) is a common method of manufacturing components from metal powders [35,36]. PBF-LB/M is distinguished by the selective sintering of successive powder layers with the use of a high-power fibre laser. It is based on the additive manufacturing of metallic materials, enabling the creation of efficient components without porosity. PBF-LB/M is becoming a method increasingly used by various industrial sectors to reduce the costs of manufacturing tools with complex shapes, especially in the case of low-volume, high-value production [36]. During the PBF-LB/M manufacturing process, metal powder layers are fully melted and fused together using thermal energy generated by a focused and computer-controlled laser energy source in accordance with the geometry of the manufactured object [37]. The sintering process is conducted in a chamber with an inert gas, most often argon or nitrogen [38]. The inert gas is designed to reduce oxidation during the process due to high temperature. Due to its growing popularity, laser powder-bed fusion may become an alternative to conventional manufacturing methods, such as machining [37,39]. PBF-LB/M includes technologies such as Direct Metal Laser Sintering (DMLS) [40], Selective Laser Melting (SLM) [41], and Direct Metal Printing (DMP) [42]. The main applications of PBF-LB/M include the production of metal parts with complex geometry that have a high strength-to-weight ratio [43,44] and the creation of complex tool geometry, including tools with cooling channels [45]. PBF-LB/M uses a high-power laser to selectively melt and bond metal powders layer by layer [46,47].

PBF-LB/M-produced components exhibit minimal surface roughness, refined microstructure, and high dimensional accuracy [48]. Advantages of PBF-LB/M technology include high solidification rates up to 10^6^ K·s^−1^ [48], efficient material utilization [49], the ability to produce components with high-resolution holes [50], and mass customization [51]. Metal powder particles are susceptible to contamination with organic substances, moisture, and oxide layers [52]. These contaminations affect the wettability of the previous layer. Thermal stresses and insufficient wettability lead to delamination and poor interlayer bonding [53]. Too much scan hatch sparing causes excessive porosity, which, according to Thijs et al. [54], can be eliminated by up to 25% scan overlap. The use of a rescanning strategy improves the surface quality [55], increases wear resistance [56], and improves densification [57].

The aim of this work is to develop and manufacture bending tools made of S235 steel with a working layer from M300 maraging steel (Steel 1.2709). PBF-LB/M was used to fabricate working layers of tools intended for the plastic forming of aluminium and nickel alloy sheets. The scope of the research work included the fabrication of M300 maraging steel tools using the PBF-LB/M method, forming of drawpieces using PBF-LB/M-produced tools, testing of tool hardness, testing of surface roughness and geometric quality of tools, testing of microhardness of drawpieces, and testing of surface roughness and geometric quality of drawpieces. The analysis of the fabrication and industrial use of PBF-LB/Med maraging steel tools will allow for the formulation of conclusions that can be the basis for strategic decisions regarding the selection of production technology in the tool industry. It will also give an answer as to whether the AM has the potential to revolutionize the conventional strategy of tool production in large-scale production.

## 2. Materials and Methods

### 2.1. Material

The material used to produce the working parts of the bending punches was the powder of the maraging tool steel M300 (Steel 1.2709) supplied by Praxair Surface Technologies (Indianapolis, IN, USA). It is a martensitic precipitation-hardened tool steel characterized by high tensile strength and hardness, good corrosion resistance, and excellent weldability. The main alloying elements of this steel are nickel, cobalt, and molybdenum. The base material for the production of the profile punches was the structural steel S235. The chemical compositions of the S235 steel and the maraging steel M300 are presented in Table 1 and Table 2, respectively. The choice was due to, on the one hand, the high mechanical properties and abrasion resistance of M300 steel, which ensure the possibility of the plastic forming of sheets without the risk of their rapid wear, and on the other hand, 235 steel being provided as the base material, as it is a relatively cheap material that also provides sufficient mechanical properties, which allows for reducing the production costs of the complete tool.

The forming of the drawpieces using additive manufactured punches was performed for 1.58 mm-thick AW-6061 T0 aluminium alloy and 0.72 mm-thick Inconel 625 Ni-based alloy sheets. The mechanical properties of the sheets (Table 3 and Table 4) were determined in a uniaxial tensile test according to the EN ISO 6892-1 [58] standard. The chemical composition of the tested sheets is presented in Table 5 and Table 6.

### 2.2. Punch Fabrication

**Figure 1:**
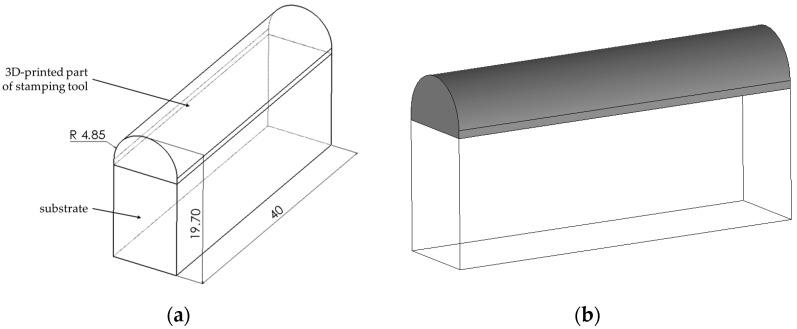
Geometry and dimensions (in mm) of the bending tool (**a**) and model of a tool part manufactured using AM (**b**).

The additive manufacturing technique by PBF-LB/M was used to produce the profile punches. The geometric model of the bending punches (Figure 1a) produced by the PBF-LB/M method was created using Solidworks software version 2016 (Dassault Systèmes, Vélizy-Villacoublay, France). The following technical parameters were taken into account: the spatial limitations of the XM200C metal 3D printer (Xact Metal, State College, PA, USA). Additionally, the specifications regarding the geometry of the punches used in simulated industrial conditions and the allowances for mechanical processing of the parts manufactured using the PBF-LB/M 3D printing technology were taken into account. The tool model (Figure 1b) was generated and saved in the STL format supported by the 3D printer.

**Figure 2:**
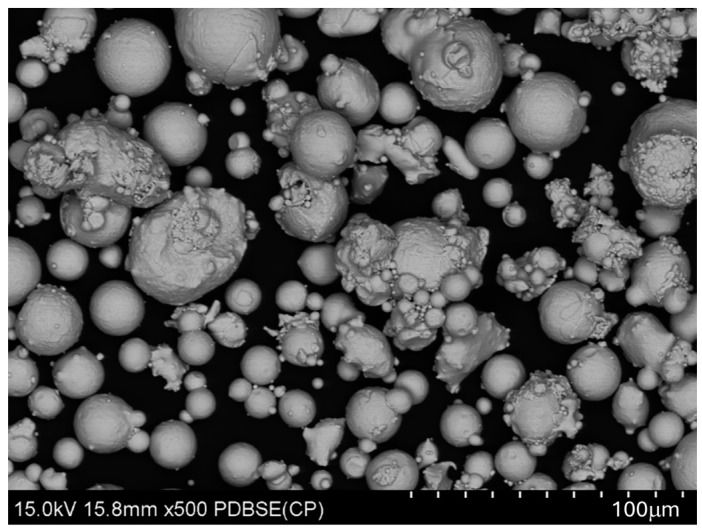
SEM image of M300 steel powder.

At the stage of technological preparation of the printing process, the Materialise Magics (Materialise, Leuven, Belgium) program dedicated to Xact Metal 3D printers was used. The solid model of the bending tool was implemented into the Materialise program. For 3D printing using the PBF-LB/M method, gas-atomized M300 maraging steel powder (Figure 2) was used, supplied by Electro Optical Systems GmbH (Krailling, Germany), together with a quality control certificate, characterized by a particle size distribution in the range of 20 to 90 μm. The specimens were 3D printed using the XM200C device (Xact Metal, State College, PA, USA). The following 3D printing parameters were set: sorting method—against gas flow, fill border offset—0.1 mm, number of exposures—2, offset transition area—0.1 mm, area tolerance—0.25 mm, and fill border offset—0.1 mm. The laser spot size was 100 microns, and the offset was set to 0.1 mm. The laser speed ranged from 250 mm·s^−1^ for the contour to 550 mm·s^−1^ for the fill. The laser power was regulated from 50 W for the contour to 80 W for the fill. The layer thickness was 0.03 mm. The bases for these settings were the guidelines proposed by the manufacturer of the 3D printer for printing powder from maraging M300 steel.

Next, the correctness of the model was verified in terms of its ability to be printed, paying special attention to aspects such as wall thickness. After making sure that the model did not require any modifications, the object was placed on a plate in the workspace of the 3D printer with dimensions of 127 × 127 × 127 mm. The program options were used to select the best arrangement. The optimal orientation allowed for the ten tool models to be spaced 0.5 mm apart in order to minimise the need for supports and improve the print quality.

**Figure 3:**
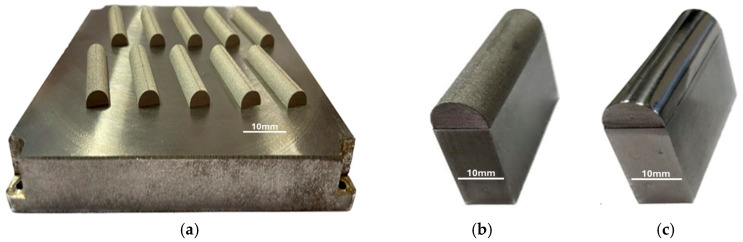
The 3D-printed M300 steel working layers of tools on a 235 steel plate (**a**), bending tool in as-received state (**b**), and bending tool after polishing (**c**).

The printing process was conducted on a previously ground and polished plate made of S235 structural steel, which was an integral part of the tool. After preparing the work platform, cleaning and evenly distributing the M300 powder, the desired objects were printed (Figure 3a). In order to minimise the oxidation process of the powder during laser sintering, the working chamber was filled with argon, which created an inert atmosphere. The final stage of printing was based on the controlled cooling of the components to prevent deformation. The finished tools were cut from the plate using a milling process, and then various types of surface treatments were applied. The punches intended for the bending process were sandblasted, ground, and polished. One of the tools was left unprocessed, in an as-received state, to serve as a reference point (Figure 3b,c).

### 2.3. Experimental Forming

**Figure 4:**
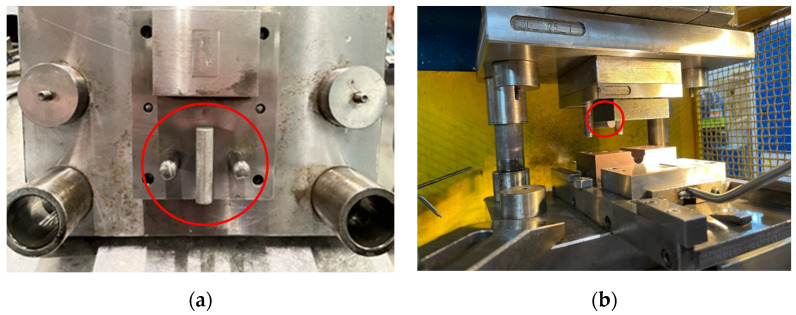
Bending tool (inside the red circle) mounted in the holder (**a**); test stand during industrial tests (**b**).

Steel tools manufactured using PBF-LB/M 3D printing technology were used during tests at ERKO sp. z o.o. sp. k. (Czeluśnica, Poland) in simulated industrial conditions of the sheet metal forming process (Figure 4). The bending process was tested using the tool in its as-received state and after polishing.

**Figure 5:**
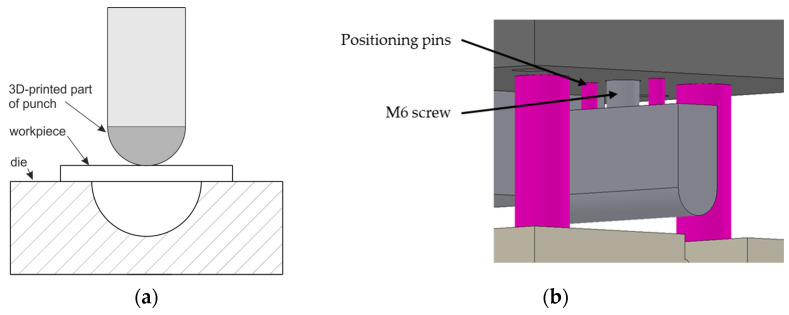
Bending process diagram (**a**); tool mounting diagram in the press (**b**).

The industrial tests included forming the AW-6061 T0 aluminium alloy and Inconel 625 nickel-based alloy sheet metals. Each of these materials was subjected to pressures of 3 Mg (29.43 kN), 5 Mg (49.05 kN), 10 Mg (98.10 kN), and 15 Mg (147.15 kN). The test samples were sheet metal strips 15.4 mm wide and 25.4 mm long. A diagram of the forming process is shown in Figure 5a, and a tool mounting diagram in the press in Figure 5b.

### 2.4. Testing of Fabricated Tools and Drawpieces

For testing tools and sheet metal elements after bending, destructive methods (microstructure, hardness, abrasion resistance) and non-destructive methods (roughness, CT, and 3D scanning) were selected, ensuring comprehensive characterization.

#### 2.4.1. Surface Topography

**Figure 6:**
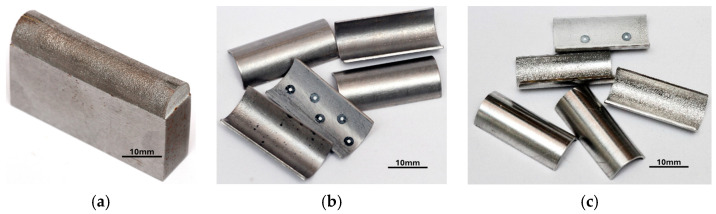
A punch with a M300 steel working part made using 3D printing technology (**a**), drawpieces made of Inconel 625 alloy sheet (**b**), and AW-6061 T0 aluminium alloy sheet (**c**).

The surface roughness and surface topography of the tools after the plastic working process (Figure 6a) and the resulting drawpieces (Figure 6b,c) were determined using the LEXT OLS 4100 (Olympus, Tokyo, Japan) laser confocal microscope. This microscope is fully automated for the observation of test objects in reflected light. It uses UV laser light with a wavelength of 405 nm.

#### 2.4.2. Tribological Tests

**Figure 7:**
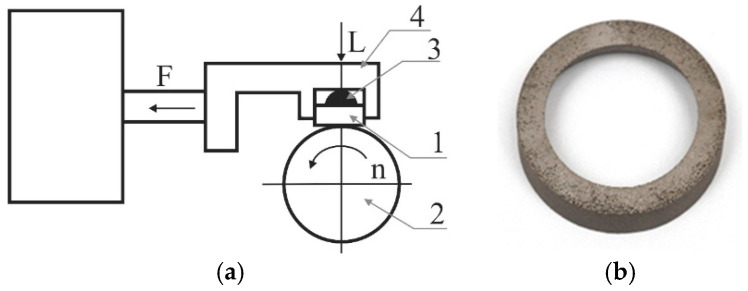
Principle of operations of T-05 roller-block tester: 1—block, 2—roller, 3—insert, and 4—sample holder (**a**) [59]; example of 3D-printed M300 steel countersample (**b**).

The abrasive wear resistance test was performed using the T-05 roller-block tester (Inst. Prec. Mech., Radom, Poland) (Figure 7a). The measurement was performed at an ambient temperature and in dry sliding friction conditions. During the tribological tests, the friction force and linear wear of the tested samples of AW-6061 T0 aluminium alloy and Inconel 625 alloy sheets were continuously registered. The M300 steel countersamples were rings with the following dimensions: internal diameter—25.0 mm, external diameter—34.7 mm, and width—9.5 mm (Figure 7b).

#### 2.4.3. Hardness Measurement

**Figure 8:**
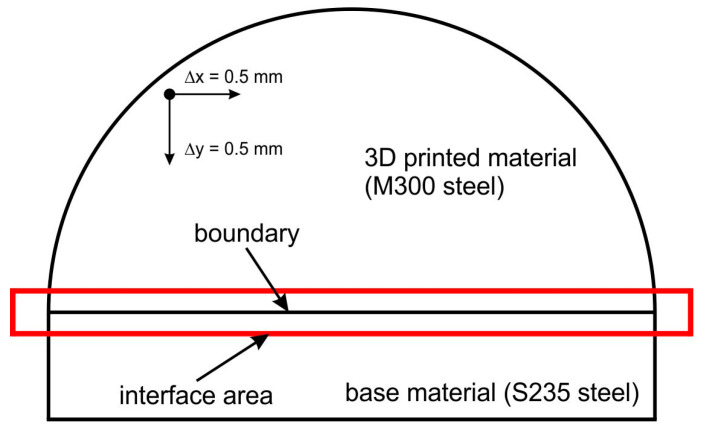
Schematic representation of Vickers hardness measurement for 3D-printed tools.

The research on the hardness of the 3D-printed punches before and after the bending process was conducted using a TUKON 2500 hardness tester (Touchstone Research Laboratory, Ltd., Triadelphia, WV, USA). The Vickers hardness was measured under a load of 1 kg (9.81 N) in accordance with the ISO 6507-1 standard [60]. In order to create a hardness distribution map in the tested samples, indentations were made at specific intervals. The indentations were made in the horizontal direction (parallel to the punch face) and vertical direction (perpendicular to the punch face). The distance between the indentations in both directions was identical and was 0.5 mm (Figure 8). Depending on the tested area, between 6 and 20 indentations were made in each row, giving a total of 232 indentations for specific punch.

In the case of drawpieces made of aluminium and nickel-based alloy sheets, which were formed using printed tools and subjected to various methods of finishing, the Vickers method was conducted on the HV1 scale. The TUKON 2500 tester was also used in the research work. The hardness tests of the drawpieces were conducted on their cross-section. The cut fragments of the drawpieces were immersed in resin, which facilitated their testing.

**Figure 9:**
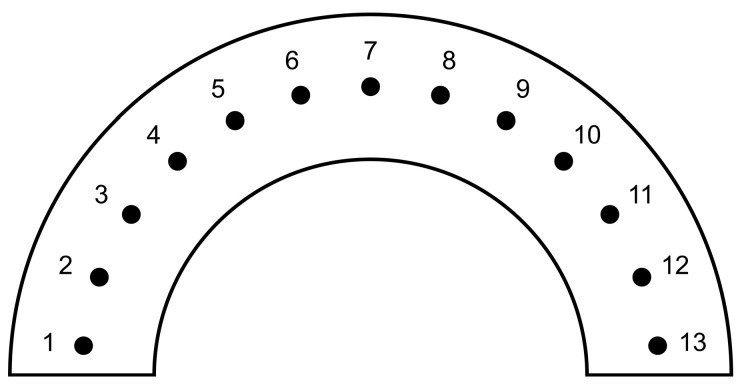
Measurement points for the hardness of drawpieces.

The measurement consisted of making a series of indentations in controlled conditions using a dedicated indenter, and then measuring the diagonals of the obtained indentations. Based on these measurements, the hardness of the tested element was calculated. Thirteen hardness measurements (Figure 9) were performed on each drawpiece.

The maximum, minimum, and average hardness values (Equation (1)) were determined for the analysed punches, and the standard deviation (Equation (2)) of these values was calculated. The arithmetic mean of the Vickers hardness measurement results was determined from the following formula:

(1) $$\bar{X}=\frac{x_{1}+x_{2}+\ldots +x_{n}}{n};$$

where *n*—number of hardness measurements.

The standard deviation is defined by the formula:

(2) $$\sigma =\sqrt{\frac{{\left(x_{1}-\bar{X}\right)}^{2}+{\left(x_{2}-\bar{X}\right)}^{2}+\ldots +{\left(x_{n}-\bar{X}\right)}^{2}}{n-1}}$$

where $x_{1},x_{2},\ldots ,x_{n}$—hardness values for *n*-measurement.

#### 2.4.4. Microstructural Analysis

The microstructures of the 3D-printed tools and the drawpieces were examined using scanning electron microscope SU 70 (Hitachi Ltd., Tokyo, Japan). The analyses of the chemical composition in the form of micro-areas of individual materials were performed using the energy dispersion spectroscopy (EDS) method. The samples were ground on abrasive paper (gradation from 200 to 4000 µm). In the next step, they were polished on polishing cloths using DIADUO (Struers, Copenhagen, Denmark) diamond pastes (gradation from 6 to 3 µm). The final processing consisted of polishing the samples using a colloidal suspension of silicon oxide. Due to the fact that the materials intended for testing were in the form of thin sheets after the stamping bending process, metallographic preparation was performed, securing the sample with an auxiliary sample. The analysis of the microstructure of the samples after the bending process was conducted using a metallo-graphic microscope Axio Vert.A1 Mat equipped with an Axiocam 305 (ZEISS, Jena, Germany) camera.

#### 2.4.5. Measurement of the Geometry of Punches and Drawpieces

The scope of the research also included geometric measurements of the punches before and after the bending process in order to assess the tool wear resulting from the production of a series of components. Accurate tool geometry measurements were performed using an Atos Core 200 (GOM a Zeiss company, Oberkochen, Germany) 3D scanner, which provided a precision of 0.02 mm. As part of the research, direct and comparative measurements were performed, which consisted of comparing and analysing 3D models. They concerned punches with different working surfaces. Additionally, measurements were also conducted on drawpieces made of AW-6061 T0 aluminium alloy and Inconel 625 alloy.

#### 2.4.6. µCT Measurements

µCT the examinations were performed using the X-ray computed tomography technique. µCT measurements were made on a Phoenix v|tomex m300 device (Waygate Technologies USA, LP, Cincinnati, OH, USA) using a microfocus lamp. The tomographic analysis process was based on three main successive tasks: the acquisition of X-ray projections, reconstruction of 3D volumes, and volumetric analysis. All samples required the use of a 0.5 mm Sn filter. The detector in each position made four measurements—one was omitted, and three were averaged. The exposure time for one measurement was set to 333 ms. The signal amplification factor was set at 0.500 on the manufacturer’s scale. In the reconstruction process, sample shifts were optimised based on the initial measurements made by the device. A shift of 0.2 px was assumed as the limit value, which allowed the study to be analysed. Each time, the virtual axis of rotation was corrected. The beam amplification corrections were applied according to the bhc + algorithm, degree 7.4. For each tool with a working layer produced by the PBF-LB/M method, porosity was determined using the VGDefX algorithm included in VG Studio MAX 3.4.1. The result of the porosity analysis are 3D images and a porosity value. The porosity value is considered to be the percentage product of the sum of the defect volume to the volume of the entire analysed element. Before starting to determine porosity, the characteristics of the absorption of materials were defined: concrete and air. The process of determining the volume of air voids was conducted on a reconstructed 3D solid. Pores whose volume exceeded 8 voxels were considered significant. The analysis revealed air voids in the entire solid or in its fragment indicated by the user. The assessment was made visually and on the basis of tables and graphs. Depending on the size of the void, the pores were shown in different colours on cross-sections and in the 3D view.

## 3. Results and Discussion

### 3.1. Testing of Punches Manufactured Using PBF-LB/M Technology

#### 3.1.1. Surface Topography of 3D-Printed Working Layer of Tools

**Figure 10:**
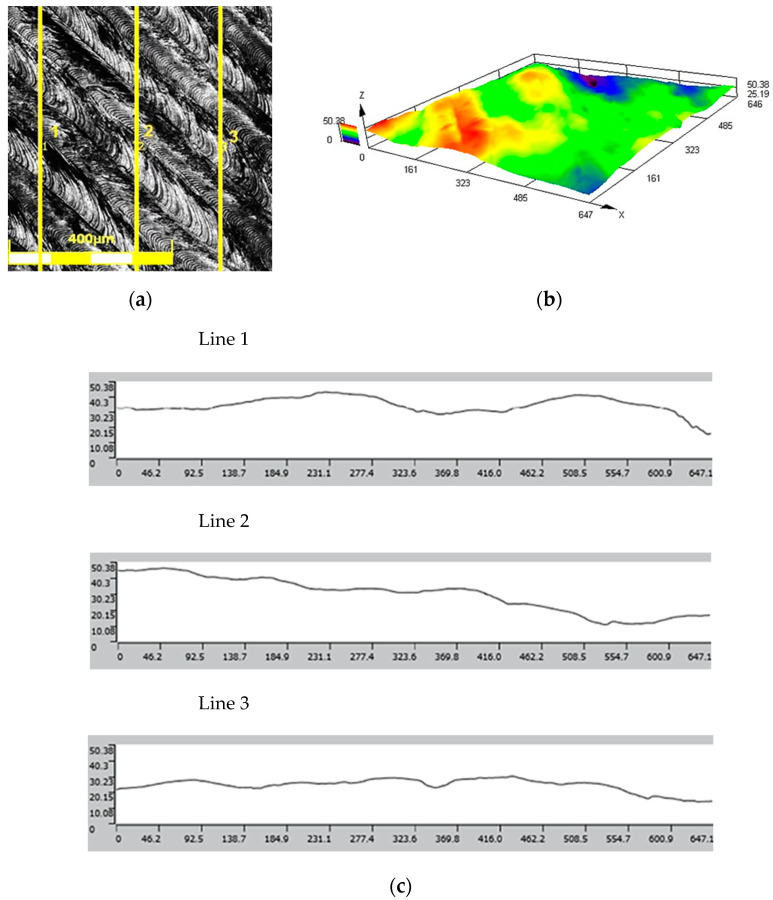
(**a**) Surface view, (**b**) surface topography, and (**c**) roughness profiles of as-received tool.

**Figure 11:**
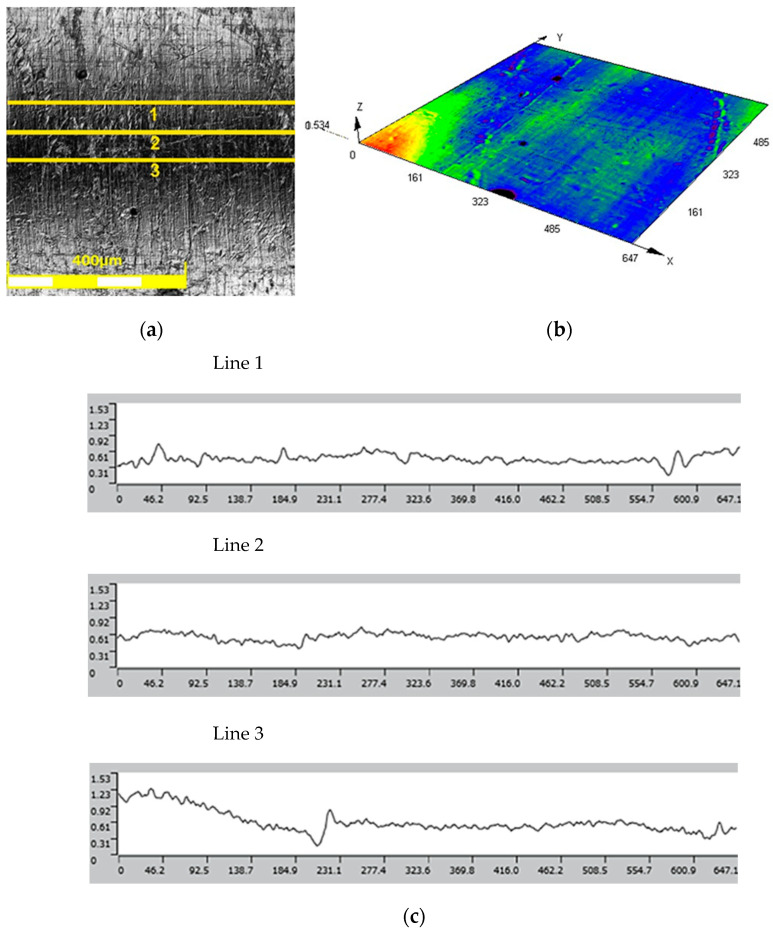
(**a**) Surface view, (**b**) surface topography, and (**c**) roughness profiles of tool after polishing.

The results of the roughness and topography measurements of the 3D-printed tools in the as-received state and after the polishing process are shown in Figure 10 and Figure 11, respectively. The surface roughness parameter values for the as-received tool are as follows.

- Mean roughness: Ra = 0.42 μm;
- Average maximum height: Rz = 28.729 μm.

The roughness class based on the above data is 8.

The values of surface roughness parameters of the polished punch are as follows.

- Mean roughness: Ra = 0.035 μm;
- Average maximum height: Rz = 0.220 μm.

The roughness class after the polishing process reached a value of up to 12.

#### 3.1.2. Wear Behaviour

**Figure 12:**
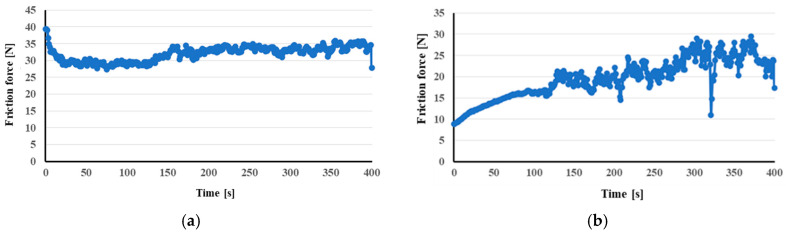
Dependence of friction force on time of tested sheet metal samples of AW-6061 T0 (**a**) and Inconel 625 (**b**).

During the testing of the AW-6061 T0 alloy sample, an increase in the ring (countersample) mass was recorded by Δm = 0.01456 g (percentage increase of 0.06%). On the other hand, the loss of mass of the AW-6061 T0 alloy sample was Δm = 0.03615 g (percentage decrease of 11.30%). This means that abrasive wear of the sample surface occurred, and its material adhered to the surface of the countersample. This phenomenon is known as galling and is related to the mechanical properties of aluminium and its alloys [61]. Filali et al. [62] related the galling aluminium alloys to the brittle nature of the oxide layer. The average value of the coefficient of friction during testing the AW-6061 T0 and M300 friction pair was μ = 0.691. The evolution of the friction force during the wear tests is shown in Figure 12. A different character of wear was observed for the Inconel 625 and M300 friction pair. During the testing of the Inconel 625 alloy sample, a loss of the ring mass was recorded by Δm = 0.03662 g (percentage decrease of 0.15%). The mass increase of the AW-6061 T0 alloy sample was Δm = 0.00024 g (percentage increase of 0.05%). The average value of the coefficient of friction during the testing of the Inconel 625 and M300 friction pair was μ = 0.518. The obtained results are consistent with the knowledge in the literature. It is well-known that the wear behaviour of metallic materials is directly related to the strength of the material and its hardness [63]. Greater material strength reduces surface wear and changes in the real contact area during the friction process [64].

#### 3.1.3. Hardness of 3D-Printed Working Layer of Tools

**Figure 13:**
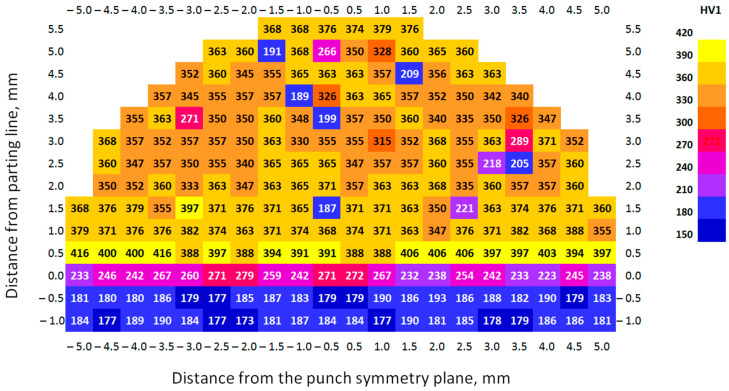
Hardness distribution on the cross-section of the PBF-LB/M printed punch—before forming.

The distribution of material hardness on the cross-section of the punch in the as-received state under industrial conditions is shown in Figure 13. Three areas were found to exist on the cross-section of the tested samples: the interface called the parting line (0 on the ordinate axis), the printed layer (above the parting line), and the substrate layer (below the parting line). In the area printed using the PBF-LB/M method, hardness values of lower than 300 HV1 were found. These are places where pores appeared in the material. Thus, they showed lower hardness than the rest of the layer obtained by 3D printing.

Table 7 shows the average hardness values determined for the 3D-printed punch before the industrial tests. They were calculated taking into account both all results and without the hardness values determined at the pore locations. Regardless of the calculation method, the printed layer was characterized by the highest average hardness values. In the case of the punch before forming, this ranged from 340 to 398 HV1, with the highest value obtained in the area at the parting line and the lowest in the middle of the printed layer. In the vicinity of the interface, the average hardness was equal to 251 HV1. The substrate of S235 structural steel itself was characterized by the lowest hardness value (183 HV1).

#### 3.1.4. Microstructural Analysis of 3D-Printed Working Layer of Tools

**Figure 14:**
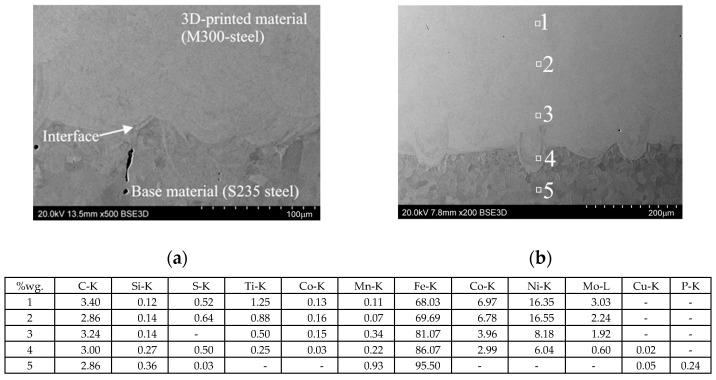
Microstructure of M300 steel after PBF-LB/M process: the cross-section of 3D-printed M300Steel and base S325 steel material (**a**) and point chemical composition of cross-section (**b**).

Microstructure studies using scanning electron microscopy have shown (Figure 14) that the use of the 3D printing method allows for the production of layers on a steel substrate. A microstructure created after 3D printing from M300 steel was observed, homogeneous across the entire width of the tested area. An uneven substrate surface is visible, which did not affect the good connection of the printed layer with the substrate. Good adhesion of the coating to the substrate was observed, which indicates good diffusion of the combined materials. An analysis of the chemical composition confirmed the presence of all expected elements in both the layer and the substrate.

#### 3.1.5. µCT Measurements of 3D-Printed Working Layer of Tools

**Figure 15:**
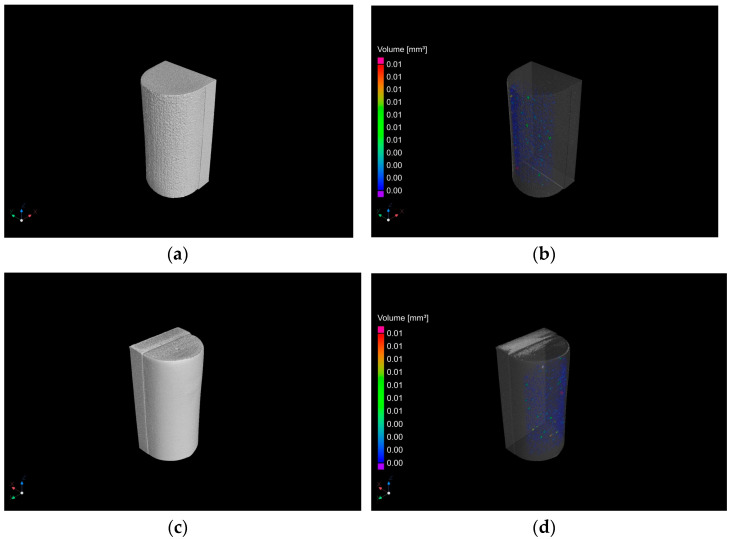
Sample with a working layer after 3D printing without surface treatment: 3D view of reconstructed data (**a**), visualization of porosity (**b**) and sample with a working layer after 3D printing and polishing 3D view of reconstructed data (**c**), and visualization of porosity (**d**).

Figure 15 shows the results of the µCT porosity measurements of the working layer of tools made by PBF-LB/M method.

The porosity analysis for samples with a working layer after 3D printing without surface treatment showed that the pores do not exceed 0.01 mm^3^ in volume, while the determined porosity is 0.23%. In the case of a sample with a working layer after 3D printing and polishing, the pores do not exceed 0.01 mm^3^, while the determined porosity is 0.34%. The samples are characterized by a small number of defects in the form of porosity. The defects occur at the surface of the working layer. Cross-sectional images indicate a greater absorption of the working layer, which is a function of the bases made of S235 steel. Several kinds of porosity (i.e., keyhole porosity, balling, lack-of-fusion, gas porosity) are characteristic for the process [65].

**Figure 16:**
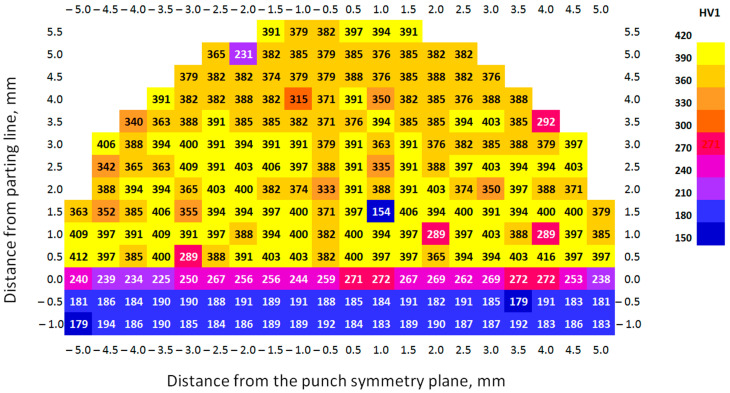
Hardness distribution on the cross-section of the PBF-LB/M printed punch (without finishing) after forming the Inconel 625 alloy sheet.

**Figure 17:**
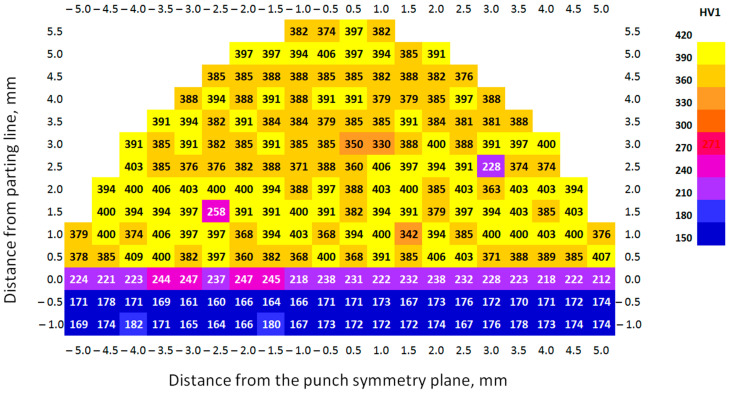
Hardness distribution on the cross-section of the PBF-LB/M printed punch (polished surface) after forming the Inconel 625 alloy sheet.

Figure 16 and Figure 17 and Table 8 show the hardness distributions on the punch cross-section after forming AW-6061 T0 and Inconel 625 sheets under simulated industrial conditions at ERKO sp. z o.o. sp. k. (Czeluśnica, Poland). Similar to the tool before shaping, hardness values of lower than 300 HV1 were found in the PBF-LB/M printed area, related to the presence of pores in the printed material.

In the case of the punches after the forming process, a similar hardness distribution was observed over the entire height of the working part of punch. The hardness of the substrate was at a comparable level, while in the interface, it ranged from 212 to 272 HV1. This difference resulted, among other things, from the location of the indentation during the measurement. Moving the measuring point towards the printed layer increased the hardness, and towards the interior of the substrate decreased it. Additionally, the parting surface was not perfectly flat and showed visible unevenness of the contact surface. In the printed layer of the punches after the sheet metal forming process, a more even distribution of average hardness was found over the height of the working part of punch, compared to the as-received punches. Similarly, a uniform hardness distribution of PBF-LB/Med samples was observed by Sun et al. [66]. In PBF-LB/M, the hardness increases non-linearly with increasing values of energy density [67]. For the Inconel 625 workpiece material, the differences between the maximum and minimum hardness values were from about 3%. However, in the case of the punch in its as-received state, this difference exceeded 12%.

### 3.2. Geometric Measurements

**Figure 18:**
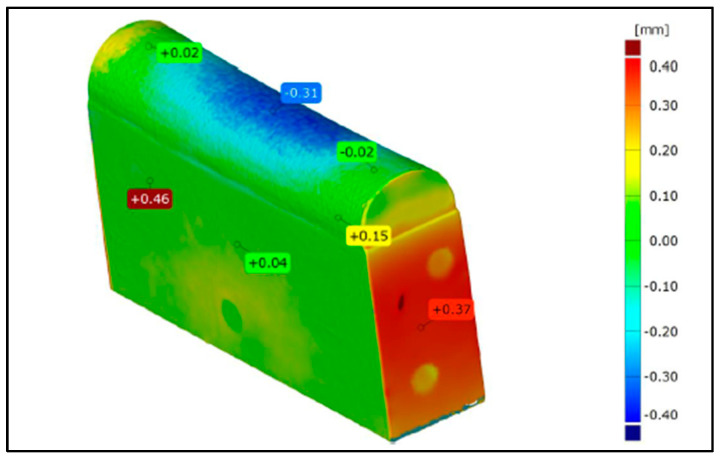
Deformation of a 3D-printed tool (without finishing) after industrial tests.

**Figure 19:**
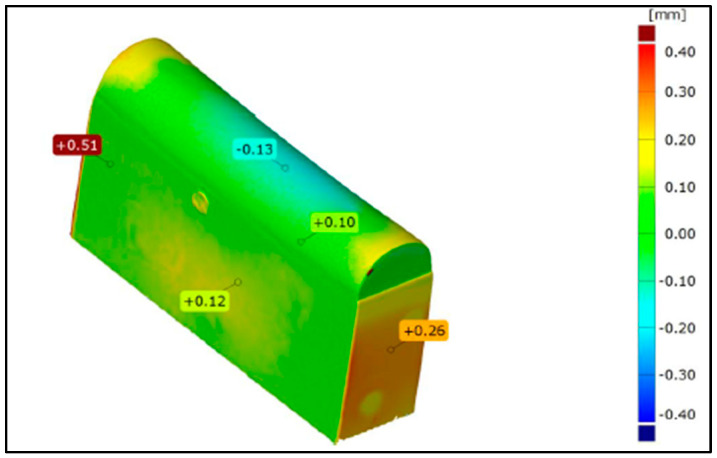
Deformation of a 3D-printed tool (polished surface) after industrial tests.

The changes in the geometry of the stamps after the forming process at characteristic points are shown in Figure 18 and Figure 19. Figure 18 illustrates the modification of the tool geometry after forming an element made of Inconel 625 alloy, while Figure 19 presents the changes for an element made of AW-6061 T0 alloy. In the case of tools manufactured using 3D printing, significant wear of the working surface was observed after industrial tests, resulting from the application of high forming forces. This phenomenon is particularly pronounced for the tool used in forming the Inconel 625 alloy element due to the much higher unit pressures required to shape the material properly. This effect was observed in both the polished tools and those without finishing treatments. As concluded by Asnafi [68] and Asnafi et al. [69], local plastic deformation and adhesive wear are main failure mechanisms of PBF-LB/M ed tools. Maraging steel, studied in this paper, is commonly used in additive manufacturing due to its high strength and resistance to dimensional changes. Dies that are 3D printed and made of maraging steel do not contain interstitial elements, which makes them suitable for the plastic working industry [70].

The tools deformed in the central part due to plastic deformation. The geometry of the tool played a significant role in the type of deformation, as the side parts of the stamp could deform freely during the process due to the absence of limiting elements. The most substantial deformation was observed in the stamp’s base, which served as the foundation for 3D printing due to its lower strength compared to the layer produced using 3D printing technology. The deformation of the printed layer largely resulted from the low strength of the substrate material.

### 3.3. Test Results of Drawpieces Made with a 3D Printing Tool

#### 3.3.1. Surface Roughness and Topography of Drawpieces

**Figure 20:**
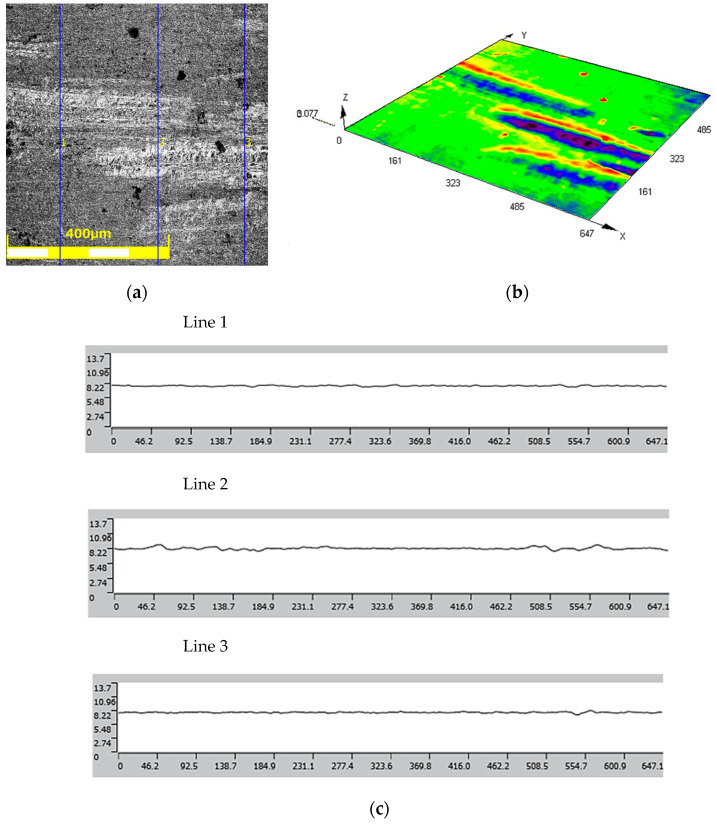
(**a**) Surface view of surface of drawpiece made of Inconel 625 material, (**b**) surface topography, and (**c**) roughness profiles.

**Figure 21:**
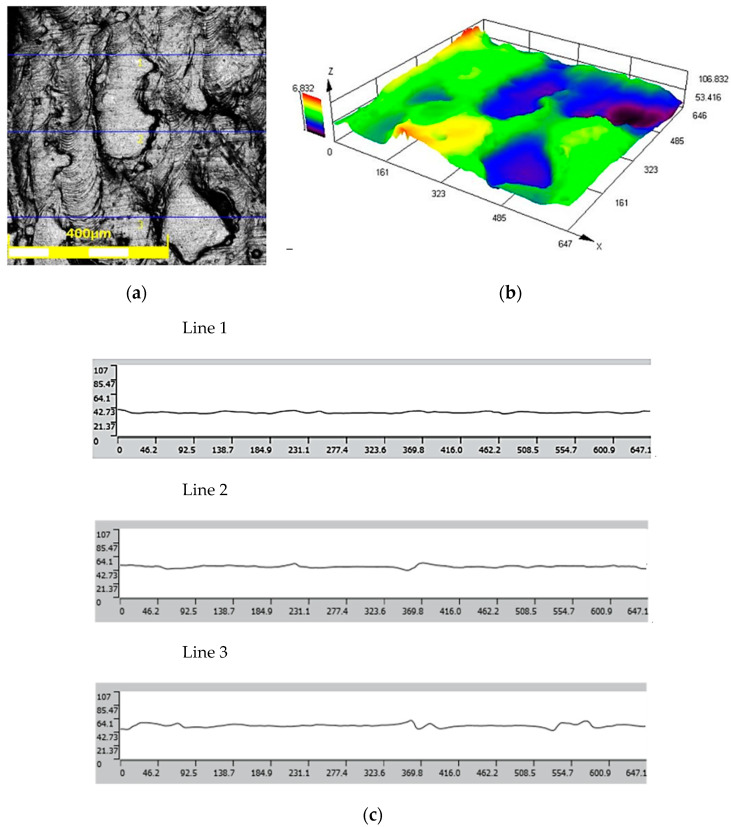
(**a**) Surface view of surface of drawpiece made of AW-6061 T0 material, (**b**) surface topography, and (**c**) roughness profiles.

The results of surface roughness measurements of the drawpieces made with 3D-printed tools from Inconel 625 are shown in Figure 20 and from AW-6061 T0 alloy in Figure 21. The average roughness value of the Inconel 625 drawpiece made with tool with a polished surface is as follows.

- Mean roughness: Ra = 0.096 μm;
- Average maximum height: Rz = 0.503 μm.

The roughness class based on the above data is 10.

The average roughness value of the Inconel 625 drawpiece made with tool with a polished surface is as follows.

- Mean roughness: Ra = 1317 μm;
- Average maximum height: Rz = 5568 μm.

The roughness class based on the above data is 12.

The results obtained by Asnafi et al. [71,72] showed that the roughness of PBF-LB/Med tools is as good as those that are conventionally made. Unfortunately, a limitation was acknowledged that the commercially available number of metallic powders for the PBF-LB/M process is limited.

#### 3.3.2. Vickers Hardness of Drawpieces

The results of the Vickers hardness measurement of the drawpieces made with the 3D-printed tool are presented in tabular form (Table 9) for all the tested samples. The maximum and minimum values were determined, as well as the average hardness measurement value (Equation (1)) and the standard deviation for each analysed sample (Equation (2)). The maximum hardness was recorded for the Inconel 625 material and the polished punch. The arithmetic mean of the hardness is similar for the same formed materials using different types of punches. However, significant differences occur in the case of the standard deviation. The highest values were observed for the Inconel 625 alloy and the polished punch.

#### 3.3.3. Microstructure of Drawpieces

**Figure 22:**
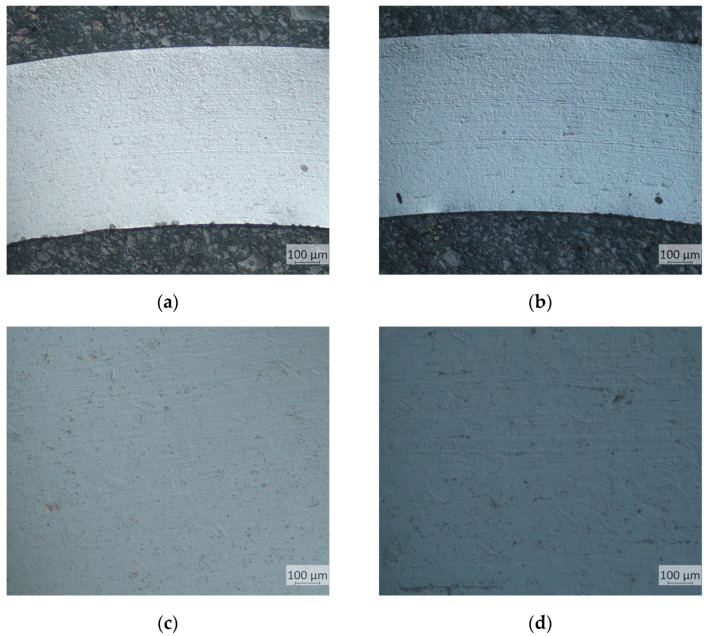
Microstructure of the Inconel 625 alloy after the stamping process: (**a**) sheet surface after forming with a PBF-LB/M printed punch, (**b**) sheet surface after forming with a polished punch, (**c**) microstructure inside the tested material after forming with a PBF-LB/M printed punch, and (**d**) microstructure inside the tested material after forming with a polished punch.

**Figure 23:**
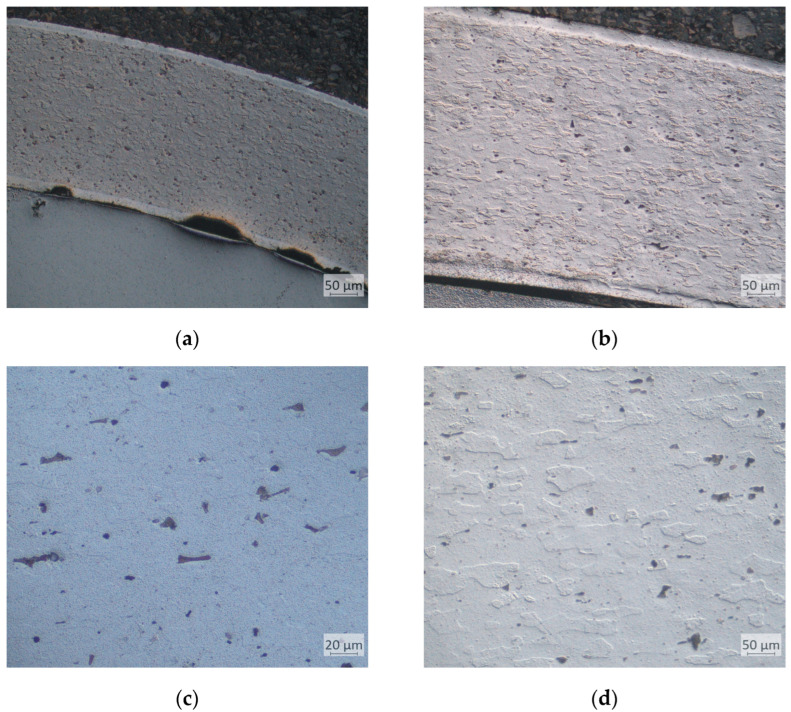
Microstructure of the AW-6061 T0 aluminium alloy after the stamping process: (**a**) sheet surface after forming with a LPBF PBF-LB/M printed punch and (**b**) sheet surface after forming with a polished punch, (**c**) microstructure inside the tested material after forming with a PBF-LB/M printed punch, and (**d**) microstructure inside the tested material after forming with a polished punch.

Microstructural tests showed an uneven surface of an aluminium alloy sheet after contact with a tool with a 3D-printed surface layer. The sheet–tool contact surface, printed and polished, did not reveal any defects in the form of material losses. This relationship was not noted in the case of nickel alloy 5599, both in the case of the printed top layer and the printed and polished one. In both cases of Inconel 625 (Figure 22) and AW-6061 T0 aluminium alloy (Figure 23), it was observed that the stamping process caused grain elongation at the point of contact between the sheet and the tool. Additionally, in the microstructure of the aluminium alloy at the point of contact between the sheet and the tool, chipping of the phases occurring in these alloys was observed.

#### 3.3.4. Geometry of Drawpieces

**Figure 24:**
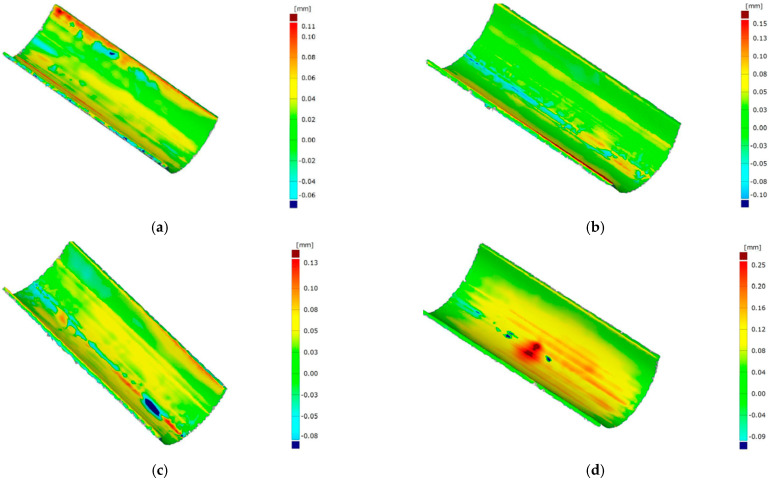
Deviation of geometry of the Inconel 625 alloy drawpieces formed under the load of: (**a**) 3 Mg (29.43 kN), (**b**) 5 Mg (49.05 kN), (**c**) 10 Mg (98.10 kN), and (**d**) 15 Mg (147.15 kN).

**Figure 25:**
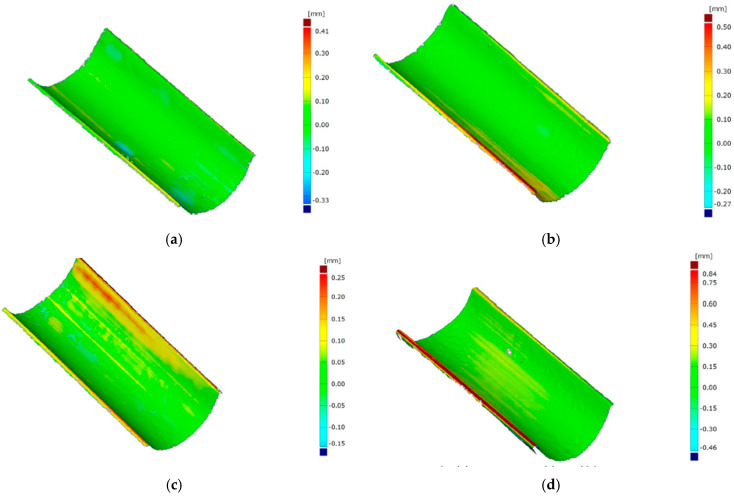
Deviation of geometry of the AW-6061 T0 aluminium alloy drawpieces formed under the load of: (**a**) 3 Mg (29.43 kN), (**b**) 5 Mg (49.05 kN), (**c**) 10 Mg (98.10 kN), and (**d**) 15 Mg (147.15 kN).

Figure 24 and Figure 25 show the results of the geometry measurements of the drawpieces. The drawpieces were produced using different loads, i.e., 3 Mg (29.43 kN), (b) 5 Mg (49.05 kN), (c) 10 Mg (98.10 kN), and (d) 15 Mg (147.15 kN). Generally, with the increase of the punch force, the value of the maximum deviations of the drawpiece shapes increases. However, these deviations are the greatest at the edge of the drawpieces. For the highest load (147.15 kN), the greatest deviations occur in the central zone of the drawpieces, which may be related to the plastic deformation of the punches. In the conventional sheet bending process, increasing the punch force reduces the elastic deformation of the sheet metal and, thus, reduces the shape deviations of the components [73,74]. In the case of drawpieces made of AW-6061 T0 aluminium alloy, for all forming forces, the greatest deviations were recorded at the edge of the drawpieces. In the remaining part, the deviations were very close in shape to the desired shape. The aluminium alloy was formed after solution heat treatment (natural aging and then cold working), which means it was more susceptible to deformation compared to Inconel 625 alloy. The mechanical properties of precipitation-hardened AW-6061 aluminium alloy are closely related to the heat treatment parameters [75]. Heat treatment affects the grain size in AW-6061 alloys, thus improving the tensile properties [76]. However, the heat treatment parameters must be precisely selected to obtain the desired formability of aluminium alloy [77].

### 3.4. Discussion

The technological process of making plastically formed parts requires a multi-stage process involving a significant amount of tooling. The tooling used must ensure repeatability and accuracy of execution. The set of tools for the plastic forming process of products for aviation and electrical engineering includes, among others, a die and a punch, which have a direct impact on the shape and quality of the part. These elements are made using standard methods through machining and electro-erosion processing. The next step requires heat treatment (hardening and tempering), and final shape processing is obtained by grinding, lapping, smoothing, or polishing. The materials used in the current technology are typical tool steels and high-speed steels for cold work such as the following.

- 1.2842 (NMV);
- 1.2379 (NC11LV);
- 1.2063 (NC6);
- Toolox 33;
- 1.7225 (40HM).

The technology of manufacturing tools from the above-mentioned materials generates significant costs of production and their service during production. Depending on the size and complexity of the detail, the CAD/CAM design and tool set (mechanical processing) takes, on average, 6 to 10 weeks. Such a long implementation time is unacceptable to customers who expect a much shorter implementation time of 2 to 4 weeks.

In addition to a short implementation time, customers also expect the following.

- Receiving a product in accordance with the specifications;
- Optimal production time;
- Lower costs of starting production to the current manufacturing technology;
- Longer service life for the equipment;
- Extension of the service intervals for the equipment;
- Reduction of service/inspection costs;
- Process repeatability;
- Respect for aspects related to environmental protection;
- Security of supply.

In plastic forming processes, one of the basic problems is the durability of tools, which, in addition to high dimensional and shape accuracy, should ensure obtaining the appropriate quality of products for the longest possible time of their operation. In the heterogeneous tool–sheet–lubricant system, there are phenomena of adhesive wear of the contact surface of the tools and the formed sheet, friction wear caused by the presence of impurities on the surface of the sheet and wear products, and fatigue wear resulting from cyclic and time-varying pressures and temperatures. Due to the complexity of the tool destruction mechanism, a quantitative description of these processes is very difficult and of little use for the needs of engineering design of plastic forming processes. A practical method of reducing excessive tool wear is the use of highly resistant protective coatings on tools and the introduction of tools made of non-metallic materials, including elastomeric and composite materials. In each of the above cases, an important role is played by technological lubricants resistant to strength and temperature, dedicated to individual process conditions. Currently used technological processes of sheet metal forming to produce parts for, among others, the aviation and automotive industries are implemented using conventional tool steels as tool material.

Due to the very wide range of production, relatively low service life of tools, and high costs of their production and regeneration, work has been undertaken to develop and implement innovative solutions using 3D metal printing to produce working parts of tools for the plastic forming of sheets made of alloys such as Al and Ni.

As part of the research, sheet forming tools were produced, consisting of a base part (base) made by machining from relatively cheap S235 steel, on which a working layer of M300 steel was produced using the 3D printing method. The shape of the base part is a cuboid, while the shape of the part (layer) made by 3D printing depends on the shape of the stamping. The 3D printing method allows for the production of any shape of the working part, and consequently, any shape of the extrusion, increasing the product portfolio, which is impossible when producing tools using the classic machining method. The main goal of the research was to determine the usability of such tools without additional heat treatment, which would contribute to the increase in the cost of tool production.

Furthermore, 3D printing technology allows for the free design of product morphology, ensuring control over product properties. An extremely important advantage of the technology used is the ability to control the morphology and texture of the layer from the level of a computer program used to design the 3D printing process. Obtaining the appropriate surface morphology will increase the oil volume in the metal–tool contact surface and, as a result, improve the tribological operating conditions during plastic forming using tools with layers applied using 3D printing. The high level of process control, including the volume of individual layers, sintering power, etc., is highly competitive with conventional methods of producing coatings for stamping tools. Layers made using the 3D metal printing method are characterized by a multi-layer structure and appropriate porosity obtained by selecting 3D printing parameters (temperature, sintering speed, amount of powder fed to the sintering zone at one time, powder grain size), ensuring an increase in the amount of lubricant in the deformation zone, reducing the coefficient of friction between the surface of the coating and the surface of the shaped material, and thus, reducing tool wear. Currently, the friction coefficient for the tool steel–Al alloy sheet metal friction pair is 0.4–0.7 when stamping without lubricant and 0.1–0.4 when stamping with lubricant, depending on the unit pressure, with constant sheet metal surface roughness. As a result, products with higher surface quality (tribological effect), increased repeatability (improved tool life and stability of process parameters), and lower manufacturing costs are obtained.

Conventional stamps, made from advanced tool steels and undergoing heat treatment, are characterized by high mechanical strength. However, their manufacturing process involves significant time and financial investments. In a direct comparison, such stamps would surpass PBF-LB/M manufactured tools in terms of strength and durability. Nonetheless, within the framework of the research, the priority was not to achieve parameters equivalent to conventional stamps, but to explore the possibility of reducing production time and costs while meeting the technical requirements for tool functionality.

The purpose of the microstructure assessment was to determine whether additional polishing of the punch surface was necessary. The punch, due to its noticeably increased roughness, could significantly affect the microstructure of the tested samples. The elevated surface roughness of the tool generated increased frictional conditions, which potentially led to local damage, microcracks, uneven deformations, and even degradation of the mechanical properties of the formed materials. Verifying the microstructure was crucial for evaluating the quality of the forming process and optimising the applied technological parameters.

The hardness test showed that in the area outside the intermediate zone between the substrate material and the printed layer, the hardness of the printed layer of the tool is distributed relatively evenly. Local reduction in hardness values occurs due to the presence of pores in these areas, most often in the central area of the punch cross-section. The hardness of the intermediate layer in all analysed cases was lower than the hardness of the printed layer and higher than the hardness of the substrate.

Additionally, measurements of the geometry of the components after the forming process were conducted using pressing forces ranging from 3 Mg (29.43 kN) to 15 Mg (147.15 kN). These analyses aimed to determine whether it was possible to achieve the correct geometry of the formed element at lower pressing forces without the need to use the maximum target force of 15 Mg (147.15 kN). Optimising the forming force was particularly important in terms of reducing tool wear and enhancing the efficiency of the production process.

The research showed that tools with a working layer directly after 3D printing, without heat treatment, show suitability for bending sheet metal in a certain range of force parameters in industrial condition without the need to perform additional operations related to surface or volume modification.

In relation to the results obtained within the conducted research, the benefits of using the working parts of tools made using 3D printing technology from metal were initially determined in relation to the tools previously made in a standard manner at ERKO, based on analyses of the times and number of operations recorded during the production of tools.

The production time is 48 h for tools with a working layer made using 3D printing and 80 h for conventional tools; the number of operations is 7 and 18, respectively, while the cost of production is about 1500 EUR and 3500 EUR, respectively. Therefore, the number of operations is over 60% lower in 3D printing technology than in the conventional method of making tool sets, and the cost of making tools using 3D printing technology from metal is over 60% lower compared to conventional production methods.

## 4. Summary and Conclusions

This paper presents the results of research on bending tools made of maraging steel manufactured using the PBF-LB/M technique. The design section of the paper was devoted to the fabrication of tools using 3D printing technology. The condition of the tool was analysed immediately after the 3D printing process was completed. The focus was on a detailed assessment of the tool after its use in simulated industrial conditions in order to determine its efficiency and durability in real operating conditions. Finally, tests were conducted on the drawpieces obtained using the additively manufactured tool. This enabled the asassessment of the efficiency and quality of the formed sheet metal elements and tool wear after 3D printing.

Based on the research results, the following conclusions were formulated.

1. As a result of the sheet metal forming process, the hardness of the punch material increased in relation to the punch before forming. This increase was at a level equal to, on average, 6%. The hardness of the printed punches made of maraging steel M300 using the PBF-LB/M method is approximately 360 HV1. The highest hardness value was measured for the punch used to form the Inconel 625 sheet (357 HV1). The highest standard deviation value of 62.80 HV1 was measured for the Inconel 625 alloy drawpiece formed using polished punch.
2. The outer surface of the tested steel is uneven, which is related to the printing process.
3. Microscopic observations of sheets after pressing using tools after 3D printing without additional processing and after polishing do not show significant differences.
4. M300 maraging steel is characterized by minimum of porosity after the printing process. An analysis of the chemical composition using the EDS method confirmed the presence of oxygen in these areas.
5. Tribological tests showed that in the case of the friction pair M300 steel and AW-6061 T0 aluminium alloy, an increase in the countersample mass by 0.06% was observed. Due to the tendency of aluminium alloys to galling, part of the worn aluminium alloy was deposited on the working surface of the countersample made of M300 steel.
6. The lowest coefficient of friction (μ = 0.518) was observed in the case of friction pair Inconel 625 and M300 steel, which explains the high share of oxides on the countersample surface and the dominant abrasive wear mechanism.
7. Geometric measurements showed that both types of tools (without finishing and after polishing), showed wear resulting from high forming force. The tools were deformed in the middle part by plastic deformation. Significantly greater deformation occurred in the part of the tool used as a substrate for 3D printing. The printed layers were deformed due to the low strength of the base material (S235 structural steel).
8. Tools fabricated using the PBF-LB/M additive manufacturing method have the potential to revolutionize tool production. Additive technologies may become more common and competitive in relation to conventional production methods, but currently, they require further development and optimisation.
9. In the future, comprehensive research is planned to explore the impact of, among others, heat treatment, surface treatment using, e.g., laser remelting or alloying, as well as the use of PVD anti-wear coatings, aimed at achieving increased hardness and wear resistance, and the scope of application of such tools, which, however, will significantly increase the costs of tool production.

## Figures and Tables

**Table 1:** Chemical composition of S235 steel (wt.%).

C	Cu	Mn	N	P	S	Fe
0.15	0.60	1.35	0.01	0.030	0.030	balance

**Table 2:** Chemical composition of maraging M300 steel (wt.%); mean value of 3 measurements.

Ni	Co	Mo	Ti	Mn	Si	C	P	S	Fe
18.50	9.75	4.25	0.90	0.10	0.10	0.01	0.005	0.005	balance

**Table 3:** Basic mechanical properties of 1.58 mm-thick AW-6061 T0 aluminium alloy sheet.

Yield Strength Rp0.2, MPa	Ultimate Tensile Strength Rm, MPa	Elongation A, %
65	115	29.0

**Table 4:** Basic mechanical properties of 0.72 mm-thick Inconel 625 alloy sheet.

Yield Strength Rp0.2, MPa	Ultimate Tensile Strength Rm, MPa	Elongation A, %
528.3	958.8	47.8

**Table 5:** Chemical composition of the AW-6061 T0 aluminium alloy (wt.%).

Mg	Mn	Fe	Si	Cu	Zn	Cr	Ti	Other	Al
1.1	0.1	0.58	0.75	0.37	0.2	0.19	0.9	0.05	95.76

**Table 6:** Chemical composition of the Inconel 625 alloy (wt.%).

Ni	Cr	Mo	Fe	Nb	Si	Al	Ti	Mn	Co	C	P	Ta	S
61.21	21.96	8.31	4.07	3.42	0.26	0.24	0.2	0.20	0.05	0.04	0.01	0.01	0.0002

**Table 7:** Distribution of average hardness values on the cross-section of the PBF-LB/M printed punch—before forming.

Distance from the Parting Line, mm	Vickers Hardness HV1	
	All Results	Without Pores
5.5	374	374
5	332	357
4.5	346	358
4	340	351
3.5	335	349
3	351	354
2.5	340	356
2	357	357
1.5	353	370
1	371	371
0.5	398	398
0	251	251
−0.5	184	184
−1	183	183

**Table 8:** Average Vickers hardness values determined for the given layers of the punch after forming (O—as-received punch; P—polished punch).

Distance from the Parting Line, mm	All Results		Without Pores	
	O	P	O	P
5.5	389		384	
5	380		395	
4.5	381		384	
4	377		388	
3.5	382		386	
3	388		384	
2.5	387		384	
2	383		396	
1.5	388		393	
1	396		389	
0.5	396		388	
0	256		230	
−0.5	187		170	
−1	187		172	

**Table 9:** Summary of the results of the hardness measurement of the drawpieces.

Sheet Material (Tool Surface Condition)	Min. HV1	Max. HV1	Arithmetic Mean HV1	Standard Deviation HV1
Inconel 625 (O)	239	321	278.23	32.24
Inconel 625 (P)	169	357	273.54	62.80
AW-6061 T0 (O)	138	149	142.46	3.15
AW-6061 T0 (P)	131	174	145.69	13.64

## Data Availability

The original contributions presented in the study are included in the article, further inquiries can be directed to the corresponding author.
